# Is There a Role for Hematopoietic Growth Factors During Sepsis?

**DOI:** 10.3389/fimmu.2018.01015

**Published:** 2018-06-21

**Authors:** Benjamin G. Chousterman, Marine Arnaud

**Affiliations:** ^1^Département d’Anesthésie-Réanimation-SMUR, Hôpitaux Universitaires Lariboisière – Saint-Louis, AP-HP, Paris, France; ^2^INSERM U1160, Hôpital Saint-Louis, Paris, France

**Keywords:** hematopoietic growth factors, G-CSF, GM-CSF, M-CSF, IL-7, IL-3, EPO, sepsis

## Abstract

Sepsis is a complex syndrome characterized by simultaneous activation of pro- and anti-inflammatory processes. After an inflammatory phase, patients present signs of immunosuppression and possibly persistent inflammation. Hematopoietic growth factors (HGFs) are glycoproteins that cause immune cells to mature and/or proliferate. HGFs also have a profound effect on cell functions and behavior. HGFs play crucial role in sepsis pathophysiology and were tested in several clinical trials without success to date. This review summarizes the role played by HGFs during sepsis and their potential therapeutic role in the Management of sepsis-related immune disturbances.

## Introduction

Infectious diseases are a major cause of death and morbidity worldwide and especially in intensive care units (ICUs) (1, 2). Decades of basic and clinical research led to the observation that most of the infections can lead to an uncontrolled response to the pathogen, the sepsis. Sepsis was recently defined by an international consortium of experts as a life-threatening organ dysfunction due to a dysregulated host response to infection (3). This definition points out that it is not only the virulence of the germ or the damages caused directly to the infected organ(s) that are responsible for the disseminated consequences on the body, it is the “host response” that is causing severe troubles. Host response is mainly mediated by the immune system. After recognition of specific patterns [from the germ, the pathogen-associated molecular patterns (PAMPs) or from the damaged cells, damage-associated molecular patterns (DAMPS)] by Pattern Recognition Receptors (PRRs), a chain reaction will lead to an auto-amplifying cytokine storm that will in turn remotely activate immune and endothelial cells (4). Organs will suffer from this friendly fire aiming to combat the initial insult. Over the last 20 years, the prognosis of septic patients has drastically improved (5). Nevertheless, there is still no specific treatment of inflammation during sepsis.

Sepsis is a complex double-face syndrome. Once having crossed the defensive barriers of the body (skin, mucus, complement, …), pathogens will activate the innate immune system and induce inflammation. As seen in most of biological processes, inflammation is well balanced by a counter-inflammation process driven by cellular reprogramming and anti-inflammatory cytokines (6, 7). The most prominent actors of the innate immunity involved in sepsis are the neutrophils and the monocytes/macrophages/dendritic cell (DC) system. Although T and B lymphocytes, usually described as part of the adaptive immune system, are also involved, they appear to play a major role in the second immunosuppressive state (sepsis-induced immunosuppression, SIS).

Indeed, after the initial phase of cell activation, the immune system appears to be “blunted” by the assault and patients face an immunosuppressive state. Poor response to infection, lymphopenia, and decreased reparative properties of immune cells are observed. Some authors termed this phase a persistent inflammation, immunosuppression, and catabolism syndrome (PICS) (8, 9). There is a debate whether the second phase clinical presentation is mostly due to inflammation or immunosuppression (10–14).

This whole sequence is compartmentalized in space and time. Immune disturbances and time variations are observed in the whole body, the hematopoietic organs (bone marrow, spleen), the blood and the tissues.

During sepsis, immune cells undergo profound phenotypic modifications in their activation state, response to stimuli, localization, and numbers. These phenomena are finely regulated by various cytokines and hematopoietic growth factors (HGFs).

An HGF is defined as a relatively stable, secreted, or membrane-bound glycoprotein that causes immune cells to mature and/or proliferate. They also have profound effects on cell functions and behaviors.

Hematopoietic growth factors are deeply involved in sepsis pathophysiology both in the initial phase and the late phase. They were naturally identified as potential therapeutic targets to treat septic patients. However, until now, there is no evidence of clinical benefit for the use of HGFs during sepsis.

In this review, we will detail how the most studied HGFs are involved in sepsis, explore the findings from clinical trials, and discuss the perspectives for HGF-mediated immunotherapy of sepsis.

## Granulocyte Macrophage – Colony Stimulating Factor (GM-CSF)

Granulocyte macrophage – colony stimulating factor (also known as CSF-2) was discovered after observing that a factor present in lung-conditioned medium was able to induce the formation of granulocytes and macrophages (15, 16). GM-CSF is coded by the Csf2 gene located on chromosome 5 in humans. GM-CSF is a monomeric glycoprotein composed of 144 amino acids [22 kilodaltons (kDa)]. GM-CSF is produced at low level during steady state and is dramatically increased during inflammatory conditions (17); it is secreted by a wide variety of cell such as monocytes-macrophages, T and B cells, mast cells, fibroblasts, and epithelial cells. The biological activity of GM-CSF is mediated through a heterodimeric cell receptor (GM-CSF-receptor, GM-CSF-R) composed of a GM-CSF specific subunit (major binding subunit GM-R_α_) and a subunit chain that is common to interleukin (IL)-5-and IL-3-receptors (common signaling subunit β_c_). GM-CSF-R signal is mediated *via* Extracellular signal-regulated kinase (ERK) 1/2, phosphoinositide-3-kinase (PI3K), mitogen-activated protein (MAP) kinase, and Janus kinase (JAK) – signal transducer and activator of transcription (STAT) pathways. Forty years after its discovery, it appears that this protein action is far more complex than just a proliferative agent; it has pleiotropic effects ranging from cell activation, survival, differentiation, chemotaxis (18), and proliferation (19). GM-CSF is frequently prescribed in patients treated by chemotherapy in order to reduce the duration of the granulocytopenia.

Until recently, most of literature regarding the role of GM-CSF during sepsis was focused on the early inflammatory phase. In murine/rat models and human cells culture, GM-CSF modulation through antibody mediated blockade, genetic invalidation, or protein supplementation helped to understand its functions in the host response to infections.

Granulocyte macrophage – colony stimulating factor role in host defense against infection is highly complex since it acts at different phases of the host response. GM-CSF deficiency is protective in models of lethal endotoxemia (20). In contrast, in infection models using full pathogens, the absence of GM-CSF appears clearly to be detrimental. In models of bacterial (21), fungal, parasitic, or viral infections (22), the absence of GM-CSF is shown to increase mortality and tissue lesions. Alveolar macrophages from GM-CSF^−/−^ mice have reduced abilities to phagocyte and kill pathogens, have reduced Fcγ receptors (FcγR) expression, and have lower membrane expression of TLRs and subsequent lipopolysaccharide (LPS) or peptidoglycan-induced tumor necrosis factor alpha (TNFα) release. GM-CSF-deficient alveolar macrophages have markedly reduced reactive oxygen species (ROS) production and adenovirus-elicited Interferon (IFN)γ, IL-18, and IL-12 production. GM-CSF also increases the expression of scavenger receptors such as macrophage receptor with collagenous structure (MARCO) and other class A scavenger receptors (SR-As) (23, 24). These scavenger receptors interact with TLRs and are shown to limit the TLR4 response in case of infection (25). Most of these pro-inflammatory and germ-killing GM-CSF effects are mediated by the transcription factor PU.1 which is essential for GM-CSF signaling during inflammation.

In caecal ligation and puncture (CLP) model, recombinant murine (rm)GM-CSF treatment improves survival and reduces bacterial translocation (26).

Interestingly, in some studies, injection of GM-CSF or genetic invalidation did not impact mice survival after CLP whether due to a timing of administration or dose issues. Inflammatory cytokines levels are higher when GM-CSF is present or enhanced (27, 28). In a model of type-A influenza infection, in which GM-CSF global deficiency is detrimental, mice with specific expression of GM-CSF only in the lung were found to have a better outcome than wild-type mice. Overexpression of GM-CSF is associated with tissue damage revealing the need for an adequate modulation (i.e., timely compartmentalization) of GM-CSF production (29).

Regarding the late phase, proliferative capacities of monocytes during sepsis in response to GM-CSF are reduced in a time-dependent manner. Early myeloid-derived suppressor cells (MDSCs) obtained 3 days after CLP procedures produced more macrophages and DCs after GM-CSF stimulation than late MDSCs obtained 12 days after CLP (30).

Granulocyte macrophage – colony stimulating factor is shown to promote type-1 pro-inflammatory cytokines production and downregulate anti-inflammatory cytokines (IL-10, IL-4) (31). GM-CSF also promotes T-cell proliferation (32) and communication with myeloid cells in the tissues (33). During the late phase of sepsis, DCs are shown to secrete less IL-12, a pivotal cytokine necessary to induce a T-Helper (T_H_)1 response. During sepsis, GM-CSF and IFNγ treatment can restore IL-12 production by splenic DCs. Mayuzumi et al. (34) showed that IL-33 promotes the generation of DC in the bone marrow through induction of GM-CSF production. GM-CSF is therefore efficient to restore TH1 response during the late phase of sepsis.

Granulocyte macrophage – colony stimulating factor has also pro-angiogenic effects and promotes endothelial cells proliferation (35–37); thus, GM-CSF could protect endothelial cells during sepsis.

The GM-CSF receptor is downregulated in human monocytes during sepsis (38) and in human neutrophils during endotoxemia (39).

The source of GM-CSF during sepsis was unknown and thought to be mainly due to macrophages activation. In 2012, a study conducted in Swirski Lab in Boston (MA, USA) tackled this question. It appears that the main source of GM-CSF following abdominal sepsis is a new cell originating from a B1-type B cells (40). This new cell type, named innate response activator B cell (IRA B cell), appears after relocalization of peritoneal B cells into the spleen where they acquire IRA B cell features. Specific depletion of GM-CSF production in B cell using a complex model of chimeric mice demonstrated the crucial role of IRA B cells in cytokine production, bacterial clearance, organ damage, and survival. In 2014, we demonstrated that GM-CSF production by IRA B cell plays a central role in the activation of a GM-CSF-IgM axis that serves as a front line of defense against pneumonia (41). Relocalization of IRA B cells and *in situ* GM-CSF production demonstrates the importance of the spatial and temporal organization of this growth factor secretion.

It appears that a lack of GM-CSF is responsible for an immunosuppressed status which in turn could be associated with a worst outcome. Treatment with GM-CSF does not appear suitable for the initial cytokine storm-associated phase. However, immunosuppression features can be reversed by GM-CSF making this protein a potential candidate as an immune therapy for SIS.

During the immunosuppressive phase, GM-CSF was mainly tested to restore monocyte functions or monocytes-granulocytes numbers. A study by Williams et al. (42) showed that GM-CSF is able to restore, *in vitro*, monocytes functions in septic patients. GM-CSF treatment increases respiratory burst activity, integrin, and CD14 expressions. Same results are observed when AIDS patients with Mycobacterium Avium Complex bacteremia are treated with GM-CSF (43).

Reduced human leukocyte antigen (HLA)-DR in a common feature of SIS, GM-CSF was shown to be effective to increase monocyte (m)HLA-DR expression. Injection of GM-CSF is more efficient than G-CSF to restore HLA-DR levels in septic neonates (44). Intraperitoneal injection of GM-CSF in peritoneal dialysis patients induces an increase in peritoneal macrophages number, integrin expression, and cytokines and chemokines production (such as IL-6 or CCL2/MCP-1) (45). Sepsis-associated neutropenia in neonates is reverted by subcutaneous injection of 5 μg/kg/day for seven consecutive days with a direct impact on mortality (46).

A clinical trial conducted by Presneill et al. (47) showed that GM-CSF treatment improves lung function in sepsis patients treated with the growth factor compared with eight controls. In a randomized trial published in 2005, GM-CSF treatment did not improve mortality but enhanced clinical and microbial resolution of infection as well as markers of monocytes and neutrophils functions (48); of note in this trial, patients with septic shock were excluded and significant proportion of patient had an organ transplant. In a trial of 58 patients, adjunction of GM-CSF to antibiotic treatment in abdominal sepsis led to reduced length of hospitalization, infectious complications, and hospital costs (49).

The most famous trial regarding the use of GM-CSF in sepsis was conducted by Meisel et al. (50). Thirty-eight septic patients with reduced mHLA-DR expression (under 8000 AB/C) for 2 days were treated with GM-CSF (4 μg/kg/d) or placebo. After 24 h, in the GM-CSF group HLA-DR significantly increases, cytokine production related to LPS stimulation increases as well. Immune cell number (neutrophils, monocytes, T cells) increases significantly compared with the control group. Duration of mechanical ventilation is shortened in the GM-CSF treatment group but no effect on mortality could be observed. This was a key study in the field of sepsis immunotherapy since the selection of patients was not only based on the diagnosis of sepsis but also on the estimation of immunosuppression through monocyte (m)HLA-DR expression measurement. In a second paper derived from these data, Schefold et al. (51) showed that GM-CSF decreases indoleamine 2,3-dioxygenase (IDO) activity and reduces kynurenine pathway activity without affecting tryptophan levels. In a remarkable study on healthy volunteers subjected to endotoxemia, GM-CSF at a dose of 4 μg/kg/day was shown to be less effective than IFNγ to reverse feature of endotoxin tolerance such as a reduced TNFα production and increased IL-10 production after LPS stimulation (52).

Bo et al. (53) conducted a meta-analysis on GM-CSF and G-CSF treatment for sepsis. Among 12 RCT identified, only four were GM-CSF-related studies (*n* = 147 patients). Mortality was consistently found to be unchanged.

A multicenter prospective randomized controlled trial is now ongoing in France (NCT02361528).

Use of GM-CSF in sepsis studies is characterized by a small number of patients and high heterogeneity of diagnosis and clinical presentation.

To date, results of clinical trials show no benefit of GM-CSF treatment during sepsis. At least, there are no major adverse events observed after nearly 20 years of clinical use. It is insufficient to conclude in a lack of safety threat but still there is no big signal of risk and there are potential benefits. There is no definitive answer to the appropriateness of GM-CSF as an immunotherapy for certain subgroups of septic patients. The timing and the phenotype of the patients that could benefit from such treatment is to be elucidated. Due to the finely regulated GM-CSF response to sepsis in time and space, intravenous or subcutaneous injections could have reached negative results because of mixed benefic and adverse effects that could be ameliorated by adding GM-CSF in specific tissues or organs and/or timepoints.

## Granulocyte – Colony Stimulating Factor (G-CSF)

Granulocyte – colony stimulating factor, also known as CSF-3, is a 25-kDa glycoprotein coded by the Csf3 gene that is located on chromosome 17 in humans. G-CSF levels are low in steady state and rise after inflammatory stimuli (54). TNFα, IL-1, or LPS stimulation of macrophages or epithelial cells induces high levels of G-CSF production (55). T cells can also induce G-CSF production through IL-17 release. The main effects of G-CSF are to induce proliferation and differentiation but also survival of cells in the neutrophils lineage. It has effects on early progenitors such as hematopoietic stem cells and in all intermediate cells up to the mature neutrophils. G-CSF-induced neutrophil production and function has been extensively studied (56–58). G-CSF plays a central role in response to infections and in situations of aplasia or neutropenia. G-CSF also enhances neutrophils production of cytokines, production of ROS, and phagocytosis when added to other stimulati. Actions of G-CSF are mediated through its receptor, the G-CSF-R. G-CSF-R requires its homodimerization in order to be fully functional. The binding of G-CSF on G-CSF-R activates a JAK-STAT phosphorylation cascade pathway. It also involves PI3K, Akt, and MAPK. Suppressor of cytokine signaling 3 SOCS3 acts as a negative regulator.

Granulocyte – colony stimulating factor is now widely used in routine to treat or prevent chemotherapy-induced neutropenia. G-CSF treatment is recommended by experts in various clinical scenarios (59–61).

Granulocyte – colony stimulating factor exerts proliferative effects but also enhances mobilization of neutrophils in a direct and indirect chemotactic effect. Intravenous or subcutaneous injection of G-CSF is rapidly followed by a marked neutrophilia together with a release of progenitors and immature cells. G-CSF has also an impact of the generation of regulatory DCs and indirectly on T-cell populations.

Granulocyte – colony stimulating factor is also shown to finely modulate the neutrophil response to infection by reducing responsiveness of neutrophils to the chemokine CXCL2 by reducing the CXCR2 mediated intracellular signaling (62). Thus, G-CSF, a mobilizing cytokine, prevents overwhelming neutrophils invasion during infections. Genetic invalidation of the G-CSF gene in mice results in neutropenia and the subsequent increased risk of developing bacterial or fungal infections and a weakened host response to infection. The impact of G-CSF loss was tested in several mice or rat models of pneumonia or abdominal sepsis. In a mice model of *P. aeruginosa* infection, G-CSF-deficient mice have decreased survival and augmented neutrophils apoptosis while local production of cytokine remains unchanged (63).

Granulocyte – colony stimulating factor is able to correct the defect of neutrophils chemotaxis to the lung in a double-hit model of LPS instillation after CLP (64). Pretreatment with G-CSF before pneumonia induction after CLP leads to increased bacterial clearance.

Granulocyte – colony stimulating factor can partially correct the impeded host response to *Klebsiella pneumoniae* infection in MCP-1/CCL2-deficient mice (65). Mice pretreated with G-CSF before CLP have improved survival (66).

Liu et al. tried to modulate the excessive inflammation related to G-CSF treatment by blocking the increase of LPS binding protein after intraperitoneal injection of feces. This leads to a reduced neutrophils infiltration into the peritoneum but with an augmented bacterial clearance ability of local neutrophils (67).

Tanaka et al. studied the levels of G-CSF after sepsis and trauma. They found elevated G-CSF levels in both conditions except that in sepsis these increased levels remained high for a larger duration (68). Ishikawa et al. studied septic patients with relative neutropenia. High levels of G-CSF at baseline were associated with poor outcome and small/no response to G-CSF treatment (69).

Most of the prospective clinical studies of G-CSF focused on patients with pneumonia. The first randomized control trial of G-CSF in severe infections was conducted by Nelson et al. Nearly 760 patients with severe community acquired pneumonia were included. G-CSF does not have an impact on mortality or length of stay but effectively increases neutrophils counts and diminishes the rate of serious complications such as ARDS or pleural empyema (70). The same authors reproduced the study with patients having multilobar pneumonia (*n* = 480) (71). The treatment does not impact mortality and shows a possible effect on patients having bacteremia. In the second largest RCT conducted on G-CSF treatment, Root et al. show that during severe sepsis secondary to pneumonia, G-CSF treatment does not improve patients’ outcome without any significant adverse event (72).

Another trial focused on patient having nosocomial pneumonia showed no clinical benefit of G-CSF treatment but was associated with a trend lower apparition of sepsis features compared with placebo (73). A study published by Stephens et al. (74) confirms the risks associated with the use of G-CSF. This randomized controlled trial included 166 septic shock patients and allocated them to be treated with G-CSF (*n* = 83) or placebo (*n* = 83). Mortality does not differ between groups but the rate of liver dysfunction and elevation of troponin raises in the G-CSF group. Thus, G-CSF appears not only to be non-beneficial but detrimental in septic patients. These results point out the risk of increasing inflammation without precise guidance.

In the meta-analysis of the impact of G-CSF during sepsis conducted by Bo et al. (53), G-CSF appears to have overall no effects on mortality and is not associated with a significant rate of adverse events. One trial is ongoing (NCT01913938) evaluating the impact of G-CSF treatment on septic patients with cytopenia with a special interest on the occurrence of hemophagocytosis.

The limits of the described trials are nearly the same for G-CSF than for GM-CSF. The main difference is that GM-CSF is a better candidate to the reversal of various features of SIS, while G-CSF is only effective on neutrophils.

## Macrophage – Colony Stimulation Factor (M-CSF)

Macrophage – colony stimulation factor, also termed CSF-1, is an 85-kDa glycoprotein in its homodimeric (secreted) form. M-CSF also exists as a membrane bound protein. The M-CSF receptor (M-CSF-R) is coded by the c-fms protooncogene. M-CSF-R has a ligand-inducible tyrosine kinase activity. Binding of M-CSF to M-CSF-R induces a dimerization, auto-phosphorylation, and activation of the kinase activity. M-CSF is also essential during pregnancy for the development and biology of the placenta.

Macrophage – colony stimulation factor increases monocyte production of G-CSF, GM-CSF, IL-6, and TNFα after LPS stimulation (75). TNFα and GM-CSF induce M-CSF production by human monocytes (76). M-CSF is also produced by several cell types, especially endothelial cells and fibroblasts.

Macrophage – colony stimulation factor plays a fundamental role in bone homeostasis; mice lacking M-CSF are osteoporotic. Using CLP model, Ogiku et al. found that the deficit of M-CSF is associated with a decreased survival, reduced phagocytosis, and increased HMGB1 levels (77).

Several studies showed that M-CSF contributes to monocytes- and macrophages-mediated immune response and bacterial clearance in response to various pathogens (78–81).

In a model of *K. pneumoniae* pneumonia, M-CSF increases locally in the lung, promotes monocytes and macrophages survival in the lung and the liver, and enhances bacterial killing (82).

Macrophage – colony stimulation factor selectively expands CD16 + monocytes in human and primates (83).

Macrophage – colony stimulation factor added to culture of murine macrophages enhances the macrophages response to TLR4 agonists while lowering the response to TLR9 agonists (CpG) (84).

Macrophage – colony stimulation factor-elevated levels during sepsis are associated with the occurrence of hemophagocytosis and thrombopenia (85).

Macrophage – colony stimulation factor can also control DC production (86). Recently, M-CSF was found to be responsible for reduced monocytes ability to convert into DC and to respond properly to inflammatory stimuli. These effects are mediated through an epigenetic regulation of the PU.1 transcription factor (87).

To date, there was no clinical trial testing the impact of M-CSF treatment during sepsis.

## Interleukin-3 (IL-3)

Interleukin-3, also named multicolony-stimulating factor (MSF), contributes to leukocyte production, proliferation, and survival (88). IL-3 stimulates the differentiation of multipotent hematopoietic stem cells into myeloid progenitor cells or, with the addition of IL-7, into lymphoid progenitor cells. IL-3 gene is located on the chromosome 5 near the GM-CSF (Csf2) gene. It is deeply involved in the pathogenesis of asthma, allergy, or blood malignancies. Until recently, the role of IL-3 in sepsis was not investigated.

In steady state, the main sources of IL-3 are activated T-helper cells. IL-3 has important effects on macrophages-DC and mastocytes in synergy with IFNγ (89).

We recently published that published that IL-3 has a crucial role in the pathogenesis of the early phase of sepsis (90). We showed that mice lacking IL-3 were partially protected from sepsis lethality induced by a CLP.

Mechanistically, we showed that IL-3 contributed to the emergency myelopoiesis that induces a rapid increase of inflammatory (Ly6C^high^) monocytes and neutrophils in blood and increases inflammation. Surprisingly, the sources of IL-3 during sepsis are the IRA B cell making these cells a producer of two crucial HGFs during sepsis (GM-CSF and IL-3) (40).

Importantly, we showed that during human sepsis, high levels of plasma IL-3 at admission were correlated with a better survival at 28 days after sepsis in two independent cohorts of patients. IL-3 levels are associated with responsiveness to corticosteroid therapy during septic shock (91).

The study of the role of IL-3 during sepsis is at its very beginning. The roles of IL-3 during the reparative phase of sepsis in mice and human are to be elucidated. As a potent DC function regulating cytokine (92), IL-3 could be involved in functional features of SIS.

## Interleukin-7 (IL-7)

Interleukin-7is a 25-kDa glycoprotein (152 amino acids in humans) mainly produced by stroma epithelial cells of the thymus and the bone marrow. IL-7 gene is located on chromosome 8.

Interleukin-7 receptor (IL-7R) is composed of two subunits: IL-7Rα (CD127) and the common gamma chain (γc) (CD132). IL-7R is expressed on the lymphoid lineage. IL-7 effects are mediated through JAK3 activation and STAT1,2,3,5, and PI3K pathways.

The main effects of IL-7 aim to maintain lymphocytes survival. It has been recognized as a potential treatment of an HIV-related lymphopenia in a phase-II trial (93).

Sepsis is associated with a lymphopenia. All types of lymphocytes, except for the regulatory T cells, see their numbers reduced in blood and tissues. The remaining lymphocytes, essentially the T cells, present signs of immunosuppression (called T-cell exhaustion). Markers of apoptosis are elevated, while the ability to proliferate is reduced together with reduced cytokines productions.

Unsinger et al. (94) used CLP model to test impact of recombinant human (rh)IL-7 treatment. rhIL7 improved mice survival, reduced drastically lymphocytes apoptosis, and improved cytokine production, especially IFNγ. LFA-1 and VLA-4, two adhesion markers, have their expression on lymphocytes increased. Il-7 is also able to improve neutrophil mobilization and recruitment through an IL-17 and CXCL1-mediated mechanism (95). IL-7 treatment can revert lethality in a model of fungemia following abdominal sepsis in mice (96). In the same model, Shindo et al. compared the effects of IL-7 and anti-PD-1 treatments (97). IL-7 is efficient at reversing T-cell exhaustion features, while anti-PD-1 increases HLA-DR expression on macrophages and DCs. These interesting results unveil a possible role for combination of immunotherapy agents during sepsis.

Recently, Terashima et al. identified osteoblasts as a major source of IL-7 during sepsis (98). Depletion of osteoblasts or suppression of osteoblasts production of IL-7 recapitulates a lymphopenic phenotype. Parathyroid hormone, an osteoblasts activator, is effective at correcting sepsis-associated lymphopenia. In a two-hit model, CLP followed by P. Aeruginosa infection, and IL-7 improves host response and survival (99).

A human clinical study shows that IL-7 gene expression is reduced during sepsis but remains surprisingly normal during bacteremia; however, IL-7 level is unchanged (100). Another study found reduced circulating IL-7 during sepsis (101). Boomer et al. noticed a reduction in IL-7R expression on lymphocytes during sepsis (102). Soluble IL-7R (sIL-7R or sCD127) levels are higher in non-surviving septic shock patients (103). Venet et al. demonstrated the potential of IL-7 to treat T-cell exhaustion during sepsis (104). In this study, IL-7 levels are augmented in septic shock patients, while it is not correlated to survival or ICU acquired secondary infections. sCD127 levels are, there again, correlated with survival but also with nosocomial infections. IL-7 is highly efficient to promote stimulated T-cell proliferation and IFNγ production. The first “proof of concept” clinical trials on IL-7 during sepsis are conducted in USA and Europe. The two studies are twins as they share the same design. The results are much awaited to evaluate IL-7 potential as a future tool for sepsis immunotherapy (IRIS-7-A and B trials, NCT02797431 and NCT02640807).

## Erythropoietin (EPO)

Erythropoietin is a 30-KDa glycoprotein mainly secreted by the peritubular interstitial fibroblasts in the kidneys. EPO gene is located on chromosome 7. EPO binding to its receptor (Epo-R that shares the common beta-chain with the IL-3, IL-5, and GM-CSF receptors) activates JAK2 signaling and increase erythropoiesis. EPO is well known for its impact on acute and chronic anemia, especially during chronic kidney disease, hematologic disease, or after chemotherapy. EPO was originally seen as potential treatment of sepsis-associated anemia. EPO levels are usually low in critically ill patients (105) but were shown to elevate in sepsis patients (106).

It appears that EPO effects on the vascular tone and its anti-apoptotic properties could also be beneficial.

Several studies have shown anti-apoptotic effects of EPO during inflammation.

In CLP model, EPO reduces renal and pulmonary damages in mice (107). EPO is also capable to correct hypotension related to sepsis by reducing endothelial nitric oxide synthase (eNOS) synthesis and inducible (i)NOS function, and preserving G-protein Receptor Kinase (GRK)2 and alpha1D receptor expressions and functions (108). EPO demonstrates cardioprotective effects in rat model of abdominal sepsis (109). Kao et al. found that EPO activates eNOS and protect skeletal muscle microvasculature (110). EPO exerts protective effect on sepsis-associated encephalopathy (111, 112).

During endotoxemia, EPO reduces AKI through decreased apoptosis (113) and activation of the beta-common receptor (114). Other group did not find such protective effects in pigs (115) possibly due to the low dose used. EPO at low dose are detrimental in endotoxinic shock (116). This may be related to dose related response to EPO and variable sensitivity of the target cells.

Erythropoietin impedes lymphoid cell apoptosis after CLP without major effect on mortality in rats (117).

The effects of EPO treatment on critical illness-associated anemia and especially sepsis-associated anemia are debated. Two major clinical studies show conflicting results (118, 119) and a recent meta-analysis concludes that the effect on anemia is small (120). Pearl discusses the negative results of EPO in trials and suggests that the doses are insufficient (121). However, when used in brain injury patients, EPO is associated with increased thrombo-embolic events (122) that could counterbalance any beneficial effects.

One clinical trial is ongoing to test the effect of EPO on microcirculatory alterations (NCT1087450) during sepsis (Table 1).

**Table 1:** Ongoing trials on the use of hematopoietic growing factors during sepsis.

Identification	Number of patients to be included	Design of the trial	Patients	Intervention	Endpoints/remarks
NCT01913938	40	Observational study	Septic patients with cytopenia	None	Aims to evaluate if the absence of response to rhG-CSF used to treat sepsis-associated cytopenia is related to hemophagocytosis

NCT02361528	488	Randomized controlled double blinded prospective trial	ICU patients presenting a severe sepsis or a septic shock associated with a sepsis-induced immunosuppression (mHLA-DR below 8000 sites/cell)	Sargramostim 125 µg/m^2^, once per day during 5 days, by subcutaneous route	Number of patients presenting at least one ICU-acquired infection at D28 or ICU discharge

NCT02797431	16	Randomized controlled double blinded prospective trial	Septic patients with lymphopenia (below 900 lymphocytes/mm3)	Two dosing frequencies of recombinant Interleukin-7 (CYT107) (10 µg/kg once or twice a week for 4 weeks)	Number of patients with absolute lymphocyte counts increased by more than 50% from baseline at Day 42 Kinetic of immune restoration through weekly measures of Absolute Lymphocyte Counts

NCT02640807	30	Randomized controlled double blinded prospective trial	Septic patients with lymphopenia (below 900 lymphocytes/mm3)	Two dosing frequencies of recombinant Interleukin-7 (CYT107) (10 µg/kg once or twice a week for 4 weeks)	Number of patients with absolute lymphocyte counts increased by more than 50% from baseline at Day 42 Kinetic of immune restoration through weekly measures of Absolute Lymphocyte Counts

NCT1087450	29	Two phases: Prospective dose response (*n* = 9) Randomized controlled double blinded prospective trial	Septic patients	Phase 1: 3 subjects per dose at 200, 400, and 600 U/kg rHuEPO Phase 2: rHuEPO vs. placebo	Changes in sub-lingual micro-circulatory blood flow for each enrolled subject using the Orthogonal Polarization Spectral imaging

**Figure 1:**
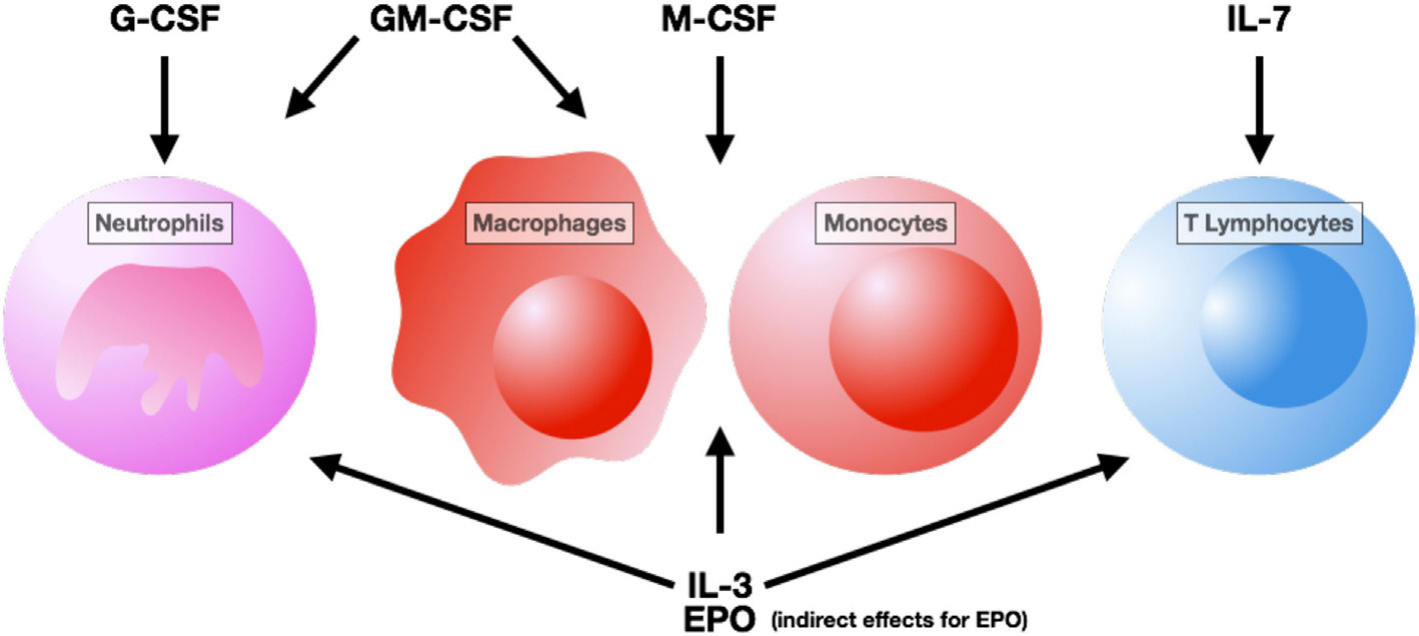
Cells targeted by hematopoietic growth factor therapy. Abbreviation: G-CSF, granulocyte – colony stimulating factor; GM-CSF, granulocyte macrophage – colony stimulating factor; M-CSF, macrophage – colony stimulating factor; IL, interleukin; EPO, erythropoietin.

**Figure 2:**
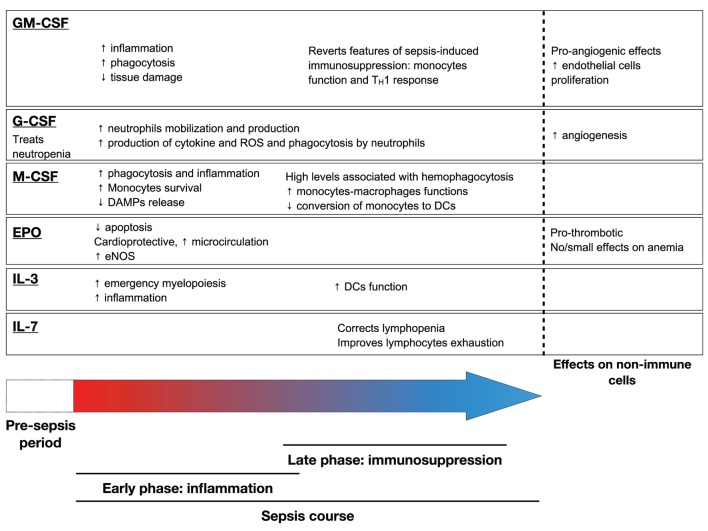
Main effects of hematopoietic growth factors during sepsis. Abbreviation: GM-CSF, granulocyte macrophage – colony stimulating factor; EPO, erythropoietin; IL-3, interleukin-3; IL-7, interleukin-7; DAMP, damage-associated molecular pattern; DC, dendritic cell; ROS, reactive oxygen species; eNOS, endothelial Nitric oxide synthase.

Figures 1 and 2 summarize the main effects of hematopoietic growth factors during sepsis.

## Conclusion

The understanding of sepsis is still limited. Tremendous efforts have been made in order to decipher its complex pathophysiology. HGFs play a crucial in both early and late phases of sepsis but there is to date no positive clinical trial regarding their use. The various pathways involved and the wide range of clinical presentation may explain partly the negatives results of clinical trials. As emphasized earlier, included patients in clinical trials are highly heterogeneous. Therefore, a potential benefit of some HGFs could have not been seen because of its futility in some clusters of patients. In addition, HGF effects are compartmentalized in space and time. Therefore, the timing of administration and the route of administration are crucial and require more developments. Most of the efforts regarding the use of HGFs during sepsis are now concentrated on the immunosuppressed patients. One important underlying question is that of the appropriateness of reintroducing inflammation in these patients. The boosting of the immune system could be exaggerated and at the end detrimental. The undergoing trial of GM-CSF and IL-7 during sepsis will help to have a better idea of their utility in this indication. EPO and G-CSF treatments seem to be deleterious during sepsis.

Anyway, there is a crucial need to be able to identify the “endotype” of sepsis patients. The biological effects of HGFs are incompletely understood and require further investigations. Next generation of sepsis trials will use this advanced knowledge and will be biomarker guided trials as recommended by experts (123). Fundamental research and clinical trial learn from each other and are complementary. In conclusion, there is no benefit with the systematic use of HGFs during sepsis, and clusters of patients that could beneficiate of such treatments are to be identified.

## Author Contributions

Both authors contributed to the literature search and the writing of the review.

## Conflict of Interest Statement

The authors declare that the research was conducted in the absence of any commercial or financial relationships that could be construed as a potential conflict of interest.
